# Mechanical and biological properties of Ti–(0–25 wt%)Nb alloys for biomedical implants application

**DOI:** 10.1093/rb/rbz042

**Published:** 2019-11-28

**Authors:** Yuqing Zhang, Danni Sun, Jun Cheng, James Kit Hon Tsoi, Jiang Chen

**Affiliations:** 1 School and Hospital of Stomatology, Fujian Medical University, Fuzhou 350002, Fujian, China; 2 Stomatological Key Laboratory of Fujian College and University, Fujian Medical University, Fuzhou 350002, Fujian, China; 3 Faculty of Dentistry, Dental Materials Science, Division of Applied Oral Sciences and Community Dental Care, The University of Hong Kong, Hong Kong SAR, China; 4 Shaanxi Key Laboratory of Biomedical Metal Materials, Northwest Institute for Non-ferrous Metal Research, Xi’an 710016, Shaanxi, China

**Keywords:** biomaterial, titanium–niobium, binary titanium alloys, low Young’s modulus, biocompatibility

## Abstract

Binary titanium–niobium (Ti–Nb) alloys have recently been attracted due to low Young’s moduli and non-toxic properties. This study explores the influence of low Nb content (0–25 wt%) on the comprehensive parameters of tensile stress–strain relationships (ultimate strength (σ_UTS_), yield strength (σ_0.2_) and elastic modulus (*E*)), surfaces properties (Vickers microhardness, surface roughness (*R*_a_), water contact angle (WCA), X-ray diffraction (XRD) and scanning electron microscopy (SEM)), corrosion resistance (in artificial saliva and lactic acid) and biological properties (cytotoxicity and alkaline phosphatase activity of MC3T3-E1 pre-osteoblasts) of Ti–*x*Nb alloys (*x* = 5, 10, 15, 20 and 25 wt%), with using commercially pure grade 2 titanium (cp-Ti) as control. XRD results shown that all the Ti–*x*Nb alloys comprised α + β Ti alloy phases, such that the β phase increased correspondingly with the increased amount of Nb in the alloy, as well as the reduction of *E* (69–87 GPa). Except Ti–5Nb, all other Ti–*x*Nb alloys showed a significantly higher hardness, increased σ_UTS_ and σ_0.2_, and decreased WCA compared with cp-Ti. No corrosion was detected on Ti–*x*Nb alloys and cp-Ti in artificial saliva and lactic acid solutions. The cytotoxicity of Ti–*x*Nb alloys was comparable to that of cp-Ti in MC3T3-E1 pre-osteoblasts without interference from differentiation behaviour, but the proliferation rate of the Ti–5Nb alloy was lower than other groups. In overall, binary Ti–(10–25 wt%)Nb alloys are promising candidate for orthopaedic and dental implants due to their improved mechanical properties and comparable biological performance, while Ti–5Nb should be used with caution.

## Introduction

Titanium (Ti) is an attractive biomaterial because of its osseointegration ability, relatively low elastic modulus and high corrosion stability. As such, Ti has received great attention in the orthopaedic and dental fields [1, 2]. However, pure Ti has insufficient wear resistance and low load-bearing strength. Thus, this hampered the use of pure titanium in long-term applications such as implant. Consequently, titanium alloys, that were commented to be more mechanically advantageous [3, 4], would be more favourable in such an application. 

Generally, Ti is a multiphase crystalline metal that is a hexagonal close-packed (α phase) structure at room temperature and can be transformed to a body-centred cubic (β phase) structure above 882°C [5, 6]. According to the lattice at room temperature, Ti alloys can be categorized as an α, a β or an α + β type. Alloying elements for Ti can be roughly divided into three categories according to their capacity to adjust the phase transformation temperatures: an α stabilizer (e.g. Al, O and N), a β stabilizer (Nb, Mo, V and Ta) and a neutral strengthening element (Zr). The properties of Ti alloys are largely dependent on the metallic composition and interior ratio of the α/β phase [7]. Ti–6Al–4V, the most widely used Ti alloys, has long been criticized for high elastic modulus and potential neurological cytotoxicity due to the dissolution of vanadium and aluminium particles [8]. Therefore, not only mechanical characteristics but also biological aspects should be considered simultaneously in the development and application of Ti alloys.

Recently, Ti–Nb alloys, which do not contain toxic elements and possess a low Young’s modulus (*E*) due to the addition of Nb as a β-stabilizer, have attracted attention. The *in vitro* and *in vivo* biological evaluation of pure metals of potential alloying elements, including Nb, Ta and Zr, indicated that they are less cytotoxic than Ti alloys or even non-toxic [9, 10]. Eisenbarth *et al.* examined the biocompatibility of pure metals of β-stabilizing elements *in vitro*, and the rank in descending order was as follows: niobium–tantalum, titanium, zirconium–aluminium [1]. Among Ti alloys, β alloys exhibit the lowest elastic modulus and wear resistance as well as a high hardenability [11]. Nb is a β-phase stabilizer that increases ratio of the β/α phase and maintains advantageous mechanical characteristics [12, 13]. The β phase in Ti–Nb alloys becomes stabilized when the ratio of valence electrons and atom numbers (e/a) is >4.2 [14]. Therefore, at least 35–40 wt% of Nb content should be included to achieve a complete and stable β phase in Ti–Nb alloys. However, since Nb is a rare metal with a high melting point, less Nb would be favoured in terms of manufacturing and cost-effectiveness considerations [15].

Some researchers also noted that martensitic Ti–Nb alloys, which have a relatively low Nb content, could have a similar elastic modulus to a Ti–Nb alloy comprising a complete β phase [16–18]. In addition, various studies have focussed on the mechanical properties of Ti–Nb binary alloys and have illustrated promising results, such as Ti–(0.1–5 wt%)Nb [19–22], Ti–(5–10 wt%)Nb [20, 21, 23, 24], Ti–(10–15 wt%)Nb [25, 26], Ti–(15–20 wt%)Nb [27, 28] and Ti–(20–25 wt%)Nb [24, 29]. Nonetheless, the biological effect of Nb content was not investigated in detail. Therefore, this study aimed to explore the influence of Nb content (0–25 wt%) on the mechanical and biological properties of Ti–Nb alloys.

## Materials and methods

### Sample preparation

The Ti–*x*Nb alloys (*x* = 5, 10, 15, 20 and 25 wt%) were the study group, and commercially pure grade 2 titanium (cp-Ti) was the control group. All specimens were provided by the Northwest Institute for Non-ferrous Metal Research (Shenyang, China). Briefly, for Ti–*x*Nb alloys, sponge titanium (99.9%) and Nb powder (99.9%) were placed in a vacuum consumable-electrode arc furnace and re-melted thrice for interior homogeneity, and then a series of forging-cogging, rolling, straightening and scaling were done. The final products were metal bars (Φ = 15 mm) that were cut into 2.0 ± 0.5 mm thick coin-like disks to suit the needs of this study. For wettability and cell behaviour studies, one side of the disks was polished sequentially with 80, 320, 600, 1000 and 2000 grit SiC papers and cleaned for 60 min with acetone, ethanol and distilled water in turn.

### Microstructural characterization

The crystallinity and phases in the Ti–*x*Nb were identified by X-ray diffraction (XRD) (Bruker Advance, USA) with CuKα radiation (40 kV, 30 mA, 10°/min) at room temperature. Phases were determined by matching the spectra to the files from the Joint Committee on Power Diffraction Standards.

### Tensile and micro-hardness tests

The tensile properties, including ultimate strength (σ_UTS_), yield strength (σ_0.2_)and elongation (*E*), were measured on an Instron 589X testing system at a crosshead speed of 15 mm/min [30]. The elastic modulus was calculated from the slope of the stress–strain curve in the elastic deformation region, and the yield strength was determined by the 0.2% offset yield method. The Vickers hardness test was performed by using an HVS-1000 digital display microhardness tester with 300 gf loading for 15 s.

### Roughness measurement

The roughness (*R*_a_) was quantified by a Surtronic 3+ (Taylor Hobson, UK). The profilometer was set to measure at every 0.8 mm cut-off value. The average of three readings of each material group was measured and recorded in triplicate on difference area.

### Corrosion behaviour test in artificial saliva and lactic acid

An immersion test that determines the release of metal elements was conducted with artificial saliva and an acid corrosive solution in accordance to ISO 10271:2011. The chemical composition of artificial saliva [31] was composed of urea (1.50 g/dm^3^), NaHCO_3_ (1.50 g/dm^3^), KCl (1.20 g/dm^3^), NaCl (0.70 g/dm^3^), KSCN (0.33 g/dm^3^), Na_2_HPO_4_ (0.26 g/dm^3^), K_2_HPO_4_ (0.20 g/dm^3^) and drops of lactic acid until the pH reached 6.72 (PHM 250, Radiometer, Denmark) to mimic the oral electrolyte [32]. The specimen was immersed in the artificial saliva with a ratio of 1 ml of solution per 1 cm^2^ of sample surface area. The solution without any metal was prepared as a blank. Finally, 4.0 ml of metallic and blank solutions were collected at 1, 7 and 28 days to determine the concentration of metallic elements released with inductively coupled plasma-optical emission spectroscopy (Spectro-Arcos) equipped with an auto-sampler. The analytical detection limit was 0.06 ppm (μg/ml), below which the concentration of the dissolved element would be considered as undetectable.

The corrosive solution included 0.1 mol/l lactic acid and 0.1 mol/l sodium chloride, and the pH was 2.21. Each alloy specimen was treated by immersing them in the corrosive solution for 7 days at 37°C, while the untreated samples were kept in the same conditions in distilled water . The corrosion resistance was determined by comparing the surface topography of the treated group and untreated group using scanning electron microscopy (SEM) (SU1510, HITACHI, Japan) together with energy dispersive spectrometry (IXRF Systems, USA) for surface chemical composition analysis.

### Water contact angle

Water contact angle (WCA) was tested by sessile drop analysis, and an auto-pipet was employed to make the same volume (10 μl) of distilled water on the suface. The drop image was stored within 1 s and analysed by a goniometer approach [33]. Then, WCA were measured in triplicate for each group (three samples per group).

### Biological evaluation

#### Cell culture

Mouse calvaria-derived MC3T3-E1 subclone 14 pre-osteoblast cells from Shanghai Institute of Biochemistry and Cell Biology (Shanghai, China) were routinely maintained in an α-MEM medium (HyClone, USA) supplemented with 10% foetal bovine serum (HyClone), 100 units/ml penicillin and 100 μg/ml streptomycin at 37°C in a 5% CO_2_ humidified atmosphere. Cells from 5 to 10 passages were used in the subsequent experiments, and cell culture medium was regularly refreshed at intervals of 2 days. For the study of pre-osteoblast differentiation, differentiation-inducing medium containing 50 μg/ml ascorbic acid and 10 mM β-glycerophosphate was added when cells reached confluence.

The Ti–*x*Nb alloy and cp-Ti disks were sterilized in an autoclave (121°C, 20 min) and dried overnight. MC3T3-E1 cells were seeded on the surface of the disks, which was placed on the plates and pre-wetted using phosphate-buffered saline (PBS) in the clean bench prior to cell research.

#### Cytotoxicity evaluation of MC3T3-E1 pre-osteoblasts

A cell viability test was conducted to measure the cytotoxicity between the studied metallic material and MC3T3-E1 pre-osteoblasts by a lactate dehydrogenase (LDH) assay and proliferation assay *in vitro*.

For evaluation of LDH activity, MC3T3-E1 cells were seeded on the surface of tested samples with a density of 1 × 10^5^/ml and incubated for 24 h at 37°C in a humidified atmosphere with 5% CO_2_. The LDH activity was determined spectrophotometrically at 490 nm according to the manufacturer’s protocol with a cytotoxicity LDH assay kit (Dojindo, Kumamoto, Japan). For metabolic activity, pre-osteoblast MC3T3-E1 was measured with resazurin-based reagent (Prestoblue^®^ Cell Viability Reagent, Life Technologies) at 24, 96 and 168 h after exposure to the material. Briefly, the cells were seeded onto specimens at a density of 2 × 10^4^/ml and incubated at 37°C in a 5% CO_2_ atmosphere. At the prescribed time, the cell culture medium was removed, replaced by a freshly prepared cell culture medium containing 10% cell viability reagent and incubated for 2 h away from direct light. The non-fluorescent resazurin was converted to highly fluorescent resorufin by live cells, which could be monitored by a colorimeter at 570 nm with 600 nm as the reference wavelength.

The performance of the Ti–*x*Nb alloys (*x* = 5, 10, 15, 20 and 25 wt%) in the LDH and proliferation assays was compared with that of the control group as a ratio (%) vs. cp-Ti (100%) to assess the difference at different times.

#### Alkaline phosphatase activity

The alkaline phosphatase (ALP) activity of pre-osteoblast MC3T3-E1 was determined at 7, 14 and 21 days with *p*-nitrophenyl phosphate (*p*NPP) after the addition of 50 μg/ml ascorbic acid and 10 mM β-glycerophosphate to the culture medium to induce differentiation. Briefly, at each time point, cells that were seeded on the Ti–*x*Nb alloys and cp-Ti were washed with PBS and incubated with trypsin/ethylenediaminetetraacetic acid for 3 min to obtain a single-cell suspension. Then, the cells were centrifuged at 1000 rpm for 10 min, and the cell pellets were collected in 1.5 ml PBS. The ALP activity was measured using the cell lysates after ultrasonic decomposition of cell pellets in accordance with the protocol provided by the manufacturer. The ALP activity was normalized by the total cellular protein content and calculated as follows:

$${\mathrm{ALP}\;\mathrm{activity}}^{*}\;=\frac{{\mathrm{OD}}_{\mathrm{sample}}-{\mathrm{OD}}_{\mathrm{blank}}}{{\mathrm{OD}}_{\mathrm{standard}}-{\mathrm{OD}}_{\mathrm{blank}}}\times \frac{\mathrm{standard}\;\mathrm{conc}}{{\mathrm{total}\;\mathrm{protein}\;\mathrm{conc}}^{**}}$$

*: the unit of ALP activity was King–Armstrong unit, which was defined as originally the amount of phosphatase that acting upon disodium phenylphosphate liberates 1 mg of phenol in 37°C in 15 min; 1 King–Armstrong unit equals 7.14 U/l.

**: total protein concentration was measured by bicinchoninic acid protein assay kit (Beyotime, China).

### Data analysis

All data were analysed by using the SPSS 19.0 software package (IBM Corporation, USA). The average values of the experiments in triplicate were expressed as the mean ±standard deviation. Multiple comparisons were conducted by one-way analysis of variance and Tukey’s post hoc test with a significance level of  0.05.

## Results

### Phases and composition

**Figure 1:**
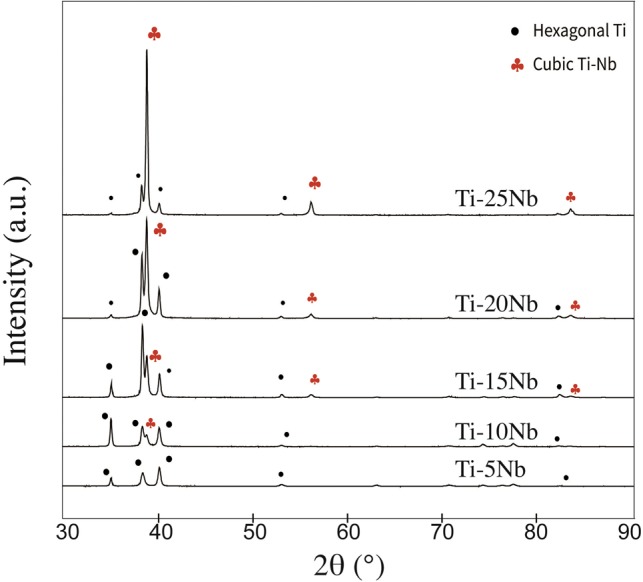
XRD spectra of Ti–*x*Nb alloys (*x* = 5, 10, 15, 20 and 25 wt%) and cp-Ti. The hexagonal α phase and cubic β phase are marked as bullets and club symbols, respectively

Figure 1 shows the XRD spectra of Ti–*x*Nb alloys (*x* = 5, 10, 15, 20 and 25 wt%). The hexagonal α phase is marked with bullets, while the cubic β phase is marked with club symbols. All Ti–*x*Nb (*x* = 5, 10, 15, 20 and 25 wt%) alloys comprised α + β Ti alloy phases, and the increase of Nb content would increase the amount of β phase. There was a very minor amount of β phase in Ti–5Nb, and Ti–10Nb exhibited a minor but detectable amount of β phase, while β phase was dominant in the Ti–25Nb, which had very little α phase.

### Mechanical properties tests

The result of roughness measurement was shown in Table 1. The roughness of cp-Ti was significantly lower than Ti–*x*Nb (*x* = 5, 10, 15, 20, 25 wt%) (*P *<* *0.05). Among Ti–*x*Nb alloys, similar roughness was observed in Ti–5Nb, Ti–10Nb and Ti–15Nb and roughness of these groups was higher than Ti–20Nb and Ti–25Nb (*P *<* *0.05). In terms of the Vickers hardness (HV5), only the hardness of the Ti–5Nb group (176.6) was lower than that of the control group (188.5), while Ti–25Nb showed the highest hardness (329.6), followed by Ti–20Nb (304.9), Ti–15Nb (249.9) and Ti–10Nb (199.82).

**Table 1:** Roughness (*R*_a_) of cp-Ti and Ti–*x*Nb (*x* = 5, 10, 15, 20 and 25 wt%)

Group	*R*_a_ (μm)
Ti–5Nb	0.34±0.02
Ti–10Nb	0.37±0.04
Ti–15Nb	0.34±0.02
Ti–20Nb	0.26±0.01
Ti–25Nb	0.27±0.01
Ti	0.25±0.01

**Figure 2:**
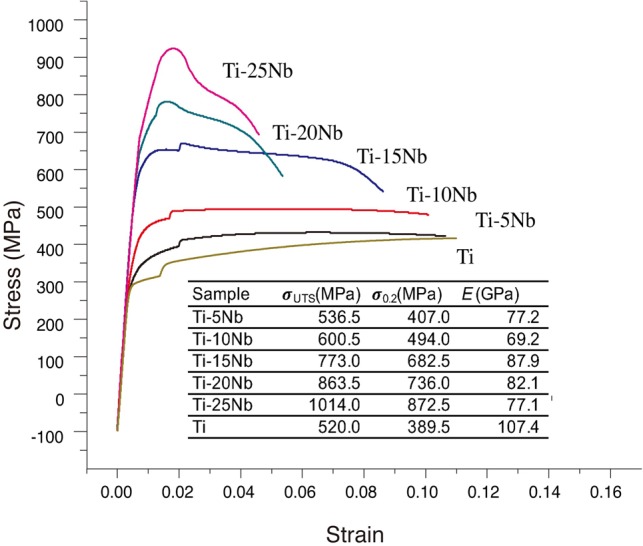
Stress–strain curves and the mechanical properties (ultimate tensile strength (σ_UTS_), yield strength (σ_0.2_) and elastic modulus (*E*)) of Ti–Nb alloys and cp-Ti

Figure 2 shows the stress–strain curves of Ti–*x*Nb alloys as well as an overview of the corresponding mechanical characteristics. In the range studied for the Ti–*x*Nb alloys, the yield strength (σ_0.2_) increased from 389.5 to 872.5 MPa as a result of the increasing Nb content; the value for cp-Ti was 389.5 MPa. In the meantime, the ability to withstand elongation due to a load, namely, the ultimate tensile strength (σ_UTS_), also increased stepwise from 520.0 to 1014 MPa.

*E* was calculated as the ratio of tensile stress to tensile strain, and there was a fluctuating trend. By adding Nb, *E* decreased, and the values of *E* for the Ti–*x*Nb alloys (*x* = 5, 10, 15, 20 and 25 wt%) were in the range of 69–88 GPa, which are significantly lower than that of cp-Ti. In this study, the elastic modulus first dropped to 69.2 GPa for the Ti–10Nb alloy and then increased to 87.9 GPa for the Ti–15Nb alloy before decreasing to 77.1 GPa for the Ti–25Nb alloy.

### Water contact angle (WCA)

**Figure 3:**
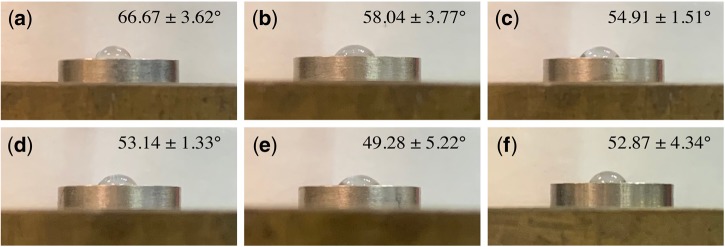
Sessile water drop measurement of (**a**) Ti–5Nb; (**b**) Ti–10Nb; (**c**) Ti–15Nb; (**d**) Ti–20Nb; (**e**) Ti–25Nb; (**f**) cp-Ti

The wetting performance was measured by evaluating the liquid–solid contact angle (CA) (Fig. 3). Compared with the cp-Ti group (52.87 ± 4.34°), the Ti–5Nb group showed a significantly lower hydrophilicity (66.67 ± 3.62°), while no statistically significant difference was observed in the other groups, which indicated a similar surface wettability to the control group. Interestingly, with an increasing mass content of Nb in Ti-Nb alloys, the average WCA showed a downward trend and the hydrophilicity was enhanced (*P* > 0.05).

### Corrosion resistance

**Figure 4:**
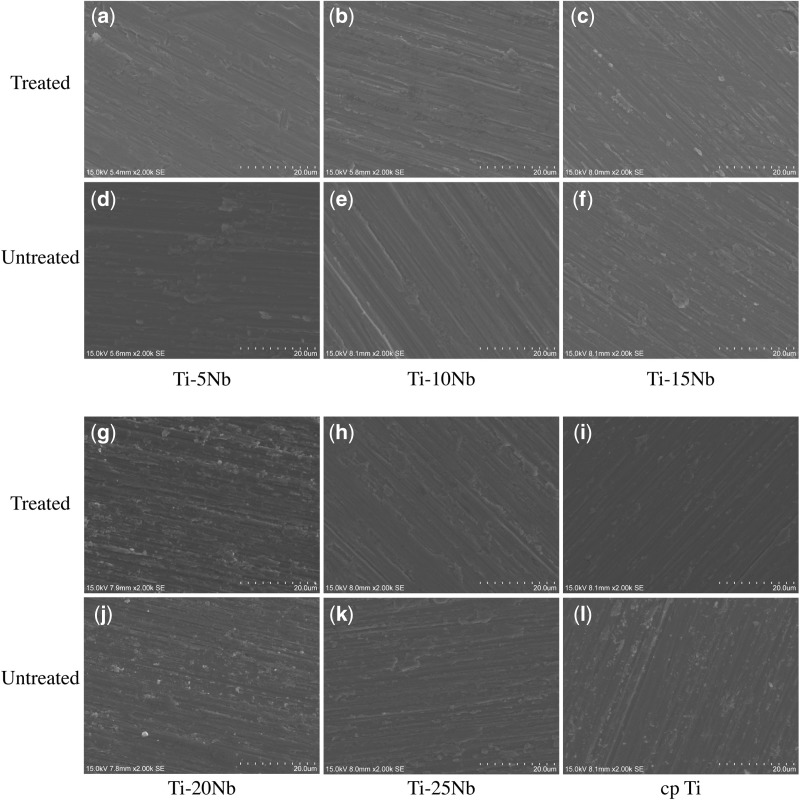
SEM topography of Ti-alloys and cp-Ti before (a–c, g–i) and after (d–f, j–l) treatment in the acid corrosive solution

The SEM topography of the Ti–*x*Nb and cp-Ti treated or untreated with lactic acid is presented in Fig. 4, and Table 2 shows the semi-quantitative chemical composition analysis by EDX None Ti and Nb elements were detected, i.e. below the detection limit (0.06 ppm), in the ICP-OES analysis on immersed solution collected at 1, 7 and 28 days. In the untreated groups, the chemical composition of Ti–*x*Nb (*x* = 5, 10, 15, 20 and 25 wt%) was roughly equivalent to that of the required mass content, with an ∼5% gradient difference between the two sequential groups. There was no difference observed in the Ti–*x*Nb alloys and cp-Ti for the Nb, Ti, O elements when comparing the lactic acid solution treatment.

**Table 2:** EDX analysis of the chemical composition of Ti–Nb alloys and cp-Ti before and after treatments with lactic acid and the subsequent corrosion

Group	Before lactic acid treatment						After lactic acid treatment					
	C	O	Na	Cl	Ti	Nb	C	O	Na	Cl	Ti	Nb
Ti–5Nb	0.36±0.01	4.78±0.18	1±0.11	1.11±0.01	85.87±0.41	6.88±0.21	0.39±0.02	4.6±0.1	1±0.03	1.17±0.06	85.99±0.12	6.84±0.11
Ti–10Nb	0.45±0.09	4.74±0.19	0.98±0.04	1.52±0.04	81.21±0.51	11.09±0.27	0.34±0.04	4.96±0.46	1.13±0.13	1.52±0.04	80.99±0.5	11.06±0.3
Ti–15Nb	0.47±0.09	4.17±0.44	1.04±0.01	1.6±0.04	77.97±0.27	14.75±0.07	0.56±0.07	3.79±0.12	1.13±0.12	1.55±0.02	78.42±0.25	14.54±0.12
Ti–20Nb	0.55±0.16	4.24±0.38	1.14±0.09	1.58±0.05	72.58±0.82	19.9±0.48	0.55±0.04	4.17±0.07	1±0.02	1.61±0.04	72.55±0.33	20.13±0.28
Ti–25Nb	0.75±0.05	4.75±0.24	1.04±0.01	1.66±0.04	67.72±0.7	24.09±0.6	0.74±0.06	4.35±0.19	1.14±0.16	1.68±0.04	67.87±0.21	24.23±0.39
cp-Ti	0.22±0.17	5.04±1.01	0.77±0.55	0.83±0.59	90.42±1.89	/	0.22±0.08	5.04±0.34	0.77±0.19	0.83±0.03	90.42±0.24	/

### Biological evaluation

#### Cytotoxicity evaluation of MC3T3-E1 pre-osteoblasts

**Figure 5:**
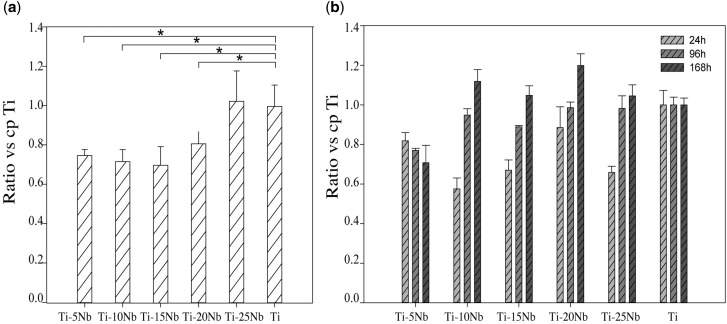
(**a**) Concentration of LDH at 24 h and (**b**) proliferation assay of MC3T3-E1 at 24, 96 and 128 h. The result was expressed as the ratio versus cp-Ti (%)

LDH, which is released during cell damage, is a common biomarker for cytotoxicity. Figure 5A shows the results of the LDH assay and that the Ti–*x*Nb alloys (*x* = 5, 10, 15, 20 and 25 wt%) demonstrated favourable biocompatibility at 24 h. The Ti–25Nb alloy exhibited a similar performance to cp-Ti (*P *>* *0.05), while the remaining Ti–*x*Nb alloys displayed a significantly lower concentration than the cp-Ti (*P *<* *0.05). The proliferation of MC3T3-E1 cells was assessed by Prestoblue^®^ Cell Viability Reagent (Fig. 5B). At 24 h, similar to the results of LDH assay, the MC3T3-E1 pre-osteoblast cells that were in contact with the Ti–5Nb, Ti–10Nb, Ti–15Nb and Ti–25Nb alloys exhibited significantly lower metabolic activity (respectively, 81.88 ± 5.08%, 7.51 ± 6.79%, 66.98 ± 3.67% and 65.88 ± 3.79%), while there was no significant difference observed for the Ti–20Nb alloy (88.55 ± 12.84%). At 96 h, there was a slight increase in the cell viability ratios of the Ti–*x*Nb alloys, except for Ti–5Nb, which showed the least proliferation (77.00 ± 3.51%). The cell viability of the Ti–10Nb and Ti–25Nb alloys exceeded 98% with values of 98.67 ± 3.51% and 98.33 ± 7.77%, respectively. At 168 h, the mean proliferation ratios of the Ti–10Nb, Ti–15Nb and Ti–25Nb alloys were similar to that of cp-Ti (*P *>* *0.05). Among the samples tested, the Ti–20Nb alloy exhibited the highest cell viability and was significantly better than that of the control group (*P *<* *0.05), while the Ti–5Nb group exhibited the lowest cell proliferation rate (*P *<* *0.05).

#### ALP activity

**Figure 6:**
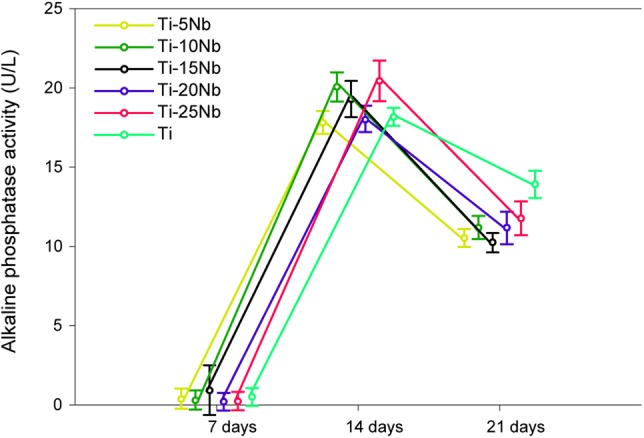
The ALP activity of MC3T3-E1 at 7, 14 and 21 days.

As one of the most recognized indicators of osteoblastic differentiation, the ALP was measured by *p*NPP at 7, 14 and 21 days, and the results are shown in Fig. 6. At 7 days, the overall ALP activity was low, and no significant difference was found between the groups. At 14 days, the ALP activity was remarkably increased, and comparable or higher ALP activity was observed in all Ti–*x*Nb alloys, among which Ti–25Nb showed the maximum ALP activity (2.89 ± 0.16), which was significantly higher than that of the cp-Ti (2.57 ± 0.06). At 21 days, the ALP activity showed a downward trend, and the performance of the Ti–*x*Nb alloys was similar (*P *>* *0.05). It is worth noting that the minimum reduction in the ALP activity occurred for the cp-Ti group (*P *<* *0.05).

## Discussion

Ti–*x*Nb (*x* = 5, 10, 15, 20 and 25 wt%) alloys were prepared, and the concentration of Nb was indirectly confirmed by using XRD. The β/α phase peak intensity ratio gradually increased with increasing Nb content. In this study, we explored the influence of Nb in Ti–*x*Nb alloys compared with cp grade 2 Ti, which are widely used [34, 35] and studied [1, 4, 36–41] in biomedical implants studies, from the following perspectives: strength, elastic modulus, corrosion resistance and biological response.

By adding Nb, even in a small amount, the tensile performance of Ti can be improved. The ultimate tensile strength and the yield strength of the Ti–5Nb alloy (536.5 and 407.0 MPa, respectively) were higher than those of cp-Ti (520 and 389.5 MPa, respectively). Hsu *et al.* also reported a higher bending strength in the Ti–5Nb group (1466 MPa) than in cp-Ti (844 MPa) [19]. Ti–6Al–4V was introduced as an implant biomaterial due to cp-Ti was suspected of inadequate strength for load-bearing devices. The ultimate tensile strength and the yield strength of Ti–25Nb alloy (1014 and 872.5 MPa, respectively) were higher than those of annealed Ti–6Al–4V (895–930 and 825–869 MPa, respectively) and could avoid the cytotoxicity effect induced by aluminium and vanadium [42]. With regards to roughness measurement, Ti–*x*Nb showed significantly higher roughness than cp-Ti group, which also indirectly reflected limited wear resistance of cp-Ti. This might prone a concern about the inflammatory response caused by wear debris and shortened longevity of implant due to component excessive wear [43–45].

In addition to the enhanced strength, the Young’s moduli decreased due to the Nb functioning as a β stabilizer in the Ti–*x*Nb alloys and was in the range of 69–87 GPa, which is remarkably lower than that of cp-Ti (*E *=* *107.4 GPa) and pure β phase Ti alloys. The study by Sumitomo *et al.* confirmed that the elastic modulus of an implant would affect bone remodelling due to Wolff’s law and that the diameter of a tibia bone in rabbits was increased for bone plates with a low rigidly [46]. Hon *et al*. separated the ‘w-shaped’ curve of Ti (14–40 mass%)–Nb alloys into three intervals, while the elastic modulus of the Ti–15Nb, Ti–20Nb and Ti–25Nb alloys was ∼92, 83 and 80 GPa, respectively [13]. Mantanni and Kudou reported that among Ti–10Nb, Ti–15Nb and Ti–20Nb alloys, the lowest *E* was found for the Ti–15Nb alloy and was reduced to 47 GPa after appropriate cooling and quenching [17]. Bonisch *et al.* studied the elastic modulus of Ti–*z*Nb (16 ≤ *z* ≤ 31 wt%) and observed a minimum *E* value for Ti–16Nb (*E *=* *60–65 GPa) [47]. Although there was a similar amount of crystalline α/β phases, the discrepancy could be attributed to a metastable phase in the Ti–Nb alloys, including close-packed α′ martensite, orthorhombic α″ and hexagonal ω, and the pattern could be generalized as *E*_β_ < *E*_α′_<*E*_α″_<*E*_ω_ [6, 48]. The overall *E* represents a complex interaction of those equilibrium and non-equilibrium phases during the manufacturing process [49]. Although all of the Ti–*x*Nb (*x* = 5, 10, 15, 20 and 25 wt%) alloys in this study comprised α + β type phases, their elastic moduli were comparable to or lower than those of some β-type Ti alloys, such as Ti–15Zr (*E *=* *112 GPa) [50], Ti–16Nb–13Ta–4Mo (*E *=* *91 GPa) [51] and Ti–15Sn–4Nb–2Ta–0.2Pd (*E *=* *89 GPa) [52]. Therefore, binary Ti–Nb alloys are still appealing for orthopaedic implants.

In terms of hydrophilicity, the WCA of the Ti–5Nb alloy was the largest among the tested alloys and significantly higher than that of cp-Ti, while the remaining alloys exhibited a WCA comparable to cp-Ti. Wetting behaviour is strongly correlated with biological responses, including adhesion of proteins, cell attachment, spreading and proliferation. In addition, hydrophilic surfaces promote higher osseointegration and bone mineralization at the early stage than hydrophobic surfaces. Ponsonnet *et al.* also reported a similar CA of cp-Ti using distilled water (53.9 ± 5.1) [4]. Luz *et al*. measured the wettability of a Ti–10Nb alloy with a PBS drop, and the CA was ∼57° [10]. A few studies have reported the relationship between wettability and the type of Ti alloy. Yu *et al.* compared the wettability of a near-α alloy (Ti–6Al–2Zr–1Mo–1V, TA15), a near-β alloy (Ti–15Mo–3Al–2.7Nb–0.2Si, TB8) and an α-β alloy (Ti–6Al–4V, TC4) [53]. The study showed that among these alloys, TC4 had the lowest CA, followed by TB8 and TA15 at different anodizing voltages. However, this result should be interpreted carefully for Ti alloys because the composition varied substantially, which may also affect the surface energy and the hydrophobicity further. Although no significant difference was observed, it was noteworthy that the WCA of Ti–*x*Nb alloys (*x* = 5, 10, 15, 20 and 25 wt%) was inversely proportional to the Nb content and positively correlated with the presence of the β phase. 

In this study, a promising anti-corrosion capacity was demonstrated in Ti–*x*Nb alloys from the measurement of the concentration of released metals, morphology and chemical composition. The concentration of metal released in the Ti–*x*Nb alloys and cp-Ti in artificial saliva for 28 days was lower than the detection limit (<0.06 μg/ml), and very few corrosion pits were found on the surface of the studied and control groups after treatment with an acid solution (pH 2.21). Furthermore, no significant difference was observed in the semi-quantitative analysis of Ti, Nb and O, and it could be concluded that the Ti–*x*Nb alloys remained stable, even in an acidic environment. The debris and metal released from implanted metallic materials are inevitable and sustainable. Upon implantation, the oral cavity, an aggressive and hostile environment to metallic alloys, stimulates an electrochemical reaction and the occurrence of corrosion [54]. The biological response of metallic implants is strongly related to their anti-corrosion properties. When Kim *et al.* immersed Ti–10Nb and Ti–20Nb alloys in Hank’s solution (pH 3.4) for 10–50 days, no metal ion release was detected (<0.01 μg/ml) and very few metal ions (0.06–0.07 μg/ml) were released after the immersion test in 0.1% lactic acid for 50 days [55]. The reason for this corrosion resistance was that Nb spontaneously formed a passive layer on the surface of the Ti–Nb-based alloys. It was different from Ti–6Al–4V, which released Al and V elements into the solution [44]. Wang *et al.* observed a stable oxide film composed of TiO_2_ and Nb_2_O_5_ and a lower passivation current density in a Ti–16Nb alloy (at%) than that in cp-Ti [56]. In addition, a recent study noted that Nb had better corrosion resistance under high levels of fluoride ions than cp-Ti, which could be ascribed to the stronger metal–metal bond strength and lower dissolution of Nb [38].

The biocompatibility between MC3T3-E1 cells and the Ti–*x*Nb surface in a short-time period was favourable in the LDH assay and proliferation test. The concentration of LDH at 24 h in all studied groups was not significantly higher than that of cp-Ti, and this result was roughly in accordance with the study of Park *et al.*, which showed that a Ti–(5, 10, 15, 20 wt%)Nb alloy demonstrated non-cytotoxicity in an agar overlay assay [57]. The proliferation curves of 24, 96 and 168 h showed an almost upward metabolic trend for the Ti–*x*Nb alloys. However, a significantly lower proliferation rate was found in the Ti–5Nb alloy at 96 and 168 h, which indicated weak cell behaviour. Among the tests, the Ti–20Nb alloy showed the highest proliferation at 168 h. Although the mean cell proliferation of the Ti–10Nb, Ti–15Nb and Ti–20Nb alloy was lower at 96 h, the performance was comparable to that of cp-Ti at 168 h. Other studies also confirmed the satisfactory biological behaviour of Nb because Ti–6Al–7Nb was a lower osteolytic mediator and had greater osteopontin synthesis and higher vinculin signals than Ti–6Al–4V [58, 59]. In addition, the current study also verified that the Ti alloy component may affect the ultimate biocompatibility. Ti–5Nb displayed a lower proliferation rate than the other groups and this phenomenon may be associated with the relatively poor wettability of Ti–5Nb.

Upon incubation in a mineralization medium for 21 days, the activity of alkaline phosphate secreted by MC3T3-E1 pre-osteoblasts was not affected by the Ti–*x*Nb alloy group and was roughly the same as that of the cp-Ti group. As an early biomarker for bone mineralization, alkaline phosphate becomes enriched when osteoblasts mature. The cellular response is extremely sensitive to the surface characteristics, and studies have discovered that variations in metallic species primarily induce alterations in matrix enzymes and ALP activity. When investigating the differentiation behaviour of human osteoblast-like cells (Saos-2) on Ti–6Al–4V or Ti–6Al–7Nb, Shapira *et al.* observed a higher expression of ALP, osteocalcin and transforming growth factor (TGFβ) for Ti–6Al–7Nb and concluded that Ti–6Al–7Nb may be more advantageous than Ti–6Al–4V for biomedical use [60]. In this study, no significant difference was observed at 7 days due to the very small amount of ALP generated. The activity of ALP dramatically increased at 14 days. There was either a similar or better performance for the Ti–*x*Nb (*x* = 5, 10, 15, 20 and 25 wt%) alloys than for cp-Ti, which demonstrated the good bone-building capacity for Nb. The activity of ALP in the cp-Ti showed a minimum reduction at 21 days and exceeded that of all the Ti–*x*Nb alloys. However, some researchers reported a similar or higher osteogenic expression in Ti–Nb alloys than that in cp-Ti after 21 days in human osteo-like MG-63 cells and mice bone marrow stromal cells (ST-2) [61].

Within the limitations of this study, in general, Ti–*x*Nb (*x* = 5, 10, 15, 20 and 25 wt%) alloys are a promising candidate for biomedical implants in terms of their mechanical properties, anti-corrosion properties, biocompatibility performance and biological behaviour. However, it should be noticed that this study only assessed the biological activities in terms of cytotoxicity of MC3T3-E1 and ALP activity. Additional experiments, such as Alizarin Red staining, PCR analysis, immune-chemistry staining and animal trials are necessary before putting Ti–Nb alloys into clinical practice. Nonetheless, Ti–5Nb should be applied with caution since the results of the WCA and proliferation rate tests were not as promising as those of other studied groups as well as cp-Ti. Further mechanical tests such as fatigue and machinability must be considered in the future.

## Conclusion

The mechanical properties of Ti–*x*Nb (*x* = 5, 10, 15, 20 and 25 wt%) depends on Nb content, due to the presence of β phase. Binary Ti–(10–25 wt%)Nb alloys are promising candidate for biomedical implants due to the improved mechanical properties and comparable biological performance, while Ti–5Nb should be used with caution.
